# Clinical and molecular landscape of surgically resected early onset pancreatic cancer

**DOI:** 10.1093/bjs/znag032

**Published:** 2026-05-07

**Authors:** Stephan B Dreyer, Adam Bryce, Fieke Froeling, Shannon Jackson, Leonor Santana, Euan J Dickson, Maria Coats, Colin McKay, David Holroyd, Andrew V Biankin, Nigel B Jamieson, David K Chang

**Affiliations:** Wolfson Wohl Cancer Research Centre, School of Cancer Sciences, University of Glasgow, Glasgow, UK; West of Scotland Pancreatic Unit, Glasgow Royal Infirmary, Glasgow, UK; Department of HPB/Transplant Surgery, Royal Infirmary of Edinburgh, Edinburgh, UK; Wolfson Wohl Cancer Research Centre, School of Cancer Sciences, University of Glasgow, Glasgow, UK; Wolfson Wohl Cancer Research Centre, School of Cancer Sciences, University of Glasgow, Glasgow, UK; Department of Oncology, Beatson West of Scotland Cancer Centre, Glasgow, UK; West of Scotland Pancreatic Unit, Glasgow Royal Infirmary, Glasgow, UK; Wolfson Wohl Cancer Research Centre, School of Cancer Sciences, University of Glasgow, Glasgow, UK; West of Scotland Pancreatic Unit, Glasgow Royal Infirmary, Glasgow, UK; West of Scotland Pancreatic Unit, Glasgow Royal Infirmary, Glasgow, UK; West of Scotland Pancreatic Unit, Glasgow Royal Infirmary, Glasgow, UK; West of Scotland Pancreatic Unit, Glasgow Royal Infirmary, Glasgow, UK; Wolfson Wohl Cancer Research Centre, School of Cancer Sciences, University of Glasgow, Glasgow, UK; West of Scotland Pancreatic Unit, Glasgow Royal Infirmary, Glasgow, UK; Wolfson Wohl Cancer Research Centre, School of Cancer Sciences, University of Glasgow, Glasgow, UK; West of Scotland Pancreatic Unit, Glasgow Royal Infirmary, Glasgow, UK; Wolfson Wohl Cancer Research Centre, School of Cancer Sciences, University of Glasgow, Glasgow, UK; West of Scotland Pancreatic Unit, Glasgow Royal Infirmary, Glasgow, UK

## Abstract

**Background:**

The increase in incidence of early-onset pancreatic cancer (EOPC) is of concern and poorly understood. The aim of this study was to investigate the clinical outcomes of surgically resected patients with EOPC and the potential molecular heterogeneity between EOPC and late age-onset disease.

**Methods:**

A retrospective cohort study was conducted, with clinical, pathological, and survival outcome data obtained from two large independent prospective cohorts curated by the Australian Pancreatic Genome Initiative (APGI) and the West of Scotland Pancreatic Unit (Glasgow Royal Infirmary) between 1997 and 2022. Patients were categorized into two age groups (<50 and ≥50 years) at time of diagnosis. Clinicopathological features and survival outcomes, in addition to gene expression and tumour microenvironment data, were compared between groups.

**Results:**

In total, 851 patients were identified, of whom 68 (8%) were aged <50 years. EOPC was associated with significantly earlier recurrence after surgery (median disease-free survival (DFS) 10.9 *versus* 14.2 months; *P* = 0.011) and there was no statistically significant difference in disease-specific survival (median 19.9 *versus* 23.8 months; *P* = 0.117). There were no differences in validated clinicopathological variables to account for the shorter DFS in the EOPC group. Despite an increased proportion of patients with EOPC receiving adjuvant chemotherapy (*P* = 0.032), DFS was significantly worse (DFS 12.6 *versus* 16.0 months; *P* = 0.022). EOPC demonstrated enrichment of genes associated with more aggressive molecular pathology and the squamous (basal-like) molecular subtype of pancreatic ductal adenocarcinoma, including *S100A2* (*P* < 0.001) and *TP63* (*P* = 0.044), and down-regulation of *GATA6* (*P* = 0.016).

**Conclusion:**

EOPC is associated with a shorter time to recurrence and more aggressive, adverse molecular pathology.

## Teaser

Early-onset pancreatic cancer (EOPC), diagnosed in patients aged <50 years, is increasing in incidence. In this study, EOPC was associated with earlier recurrence, despite increased use of adjuvant chemotherapy, and with more aggressive molecular features, which may underpin the shorter disease-free interval in EOPC patients.

## Introduction

Despite significant increases in the understanding of the molecular pathology underlying pancreatic cancer, only minor therapeutic advances have been made. There remains a lack of understanding of the development and progression of the disease and why it is so lethal. The most common form, pancreatic ductal adenocarcinoma (PDAC), is a major cancer of unmet need in Western societies with a 5 year survival that still does not exceed 10%^1–3^.

There is a concerning trend of increasing incidence of many gastrointestinal malignancies in young patients, including PDAC^4–11^. This trend is most pronounced in young women, with a worrying rise in incidence that does not seem to be slowing down^7^. The underlying cause for this cannot be explained by current data. The interaction between race, age, sex, and socioeconomic factors with risk of PDAC in early age is not understood and investigating this is challenging in what remains a low-incidence cancer, albeit with high mortality.

Younger patients are often presumed to tolerate multimodality treatment better, potentially leading to better outcomes. However, the evidence across many malignancies is conflicting, with some studies suggesting that early onset associates with more aggressive disease^6,12,13^. Improved performance status and perceived clinician preference for more aggressive treatments are believed to offer young patients a wider range of treatment options with improved survival. Yet very few studies have investigated the differences in outcomes observed for younger and older patients with PDAC. Previous studies have demonstrated mixed results regarding the outcomes of patients with early-onset PDAC (that is early-onset pancreatic cancer (EOPC)) and late age-onset PDAC^14–16^.

PDAC is believed to develop over a number of years, or even decades, before becoming clinically relevant^17^. However, the molecular factors, including genomic mutations, that are associated with EOPC are poorly understood. There have been very few studies investigating these factors and how they affect clinical outcomes.

The aim of this study was to investigate the clinical outcomes of patients with EOPC for surgically resected disease and investigate the molecular differences between EOPC and late age-onset disease.

## Methods

### Patient cohort description

Surgically resected patients were recruited from prospectively maintained databases after pancreatectomy from the West of Scotland Pancreatic Unit, Glasgow Royal Infirmary, UK and the Australian Pancreatic Cancer Genome Initiative (APGI) cohort (www.pancreaticcancer.net.au, as part of the International Cancer Genome Consortium (ICGC; www.icgc.org))^18–20^. Patients were recruited between 1997 and 2022 (Glasgow) and between 1998 and 2013 (APGI). In this study, pancreatic cancer refers specifically to PDAC, unless otherwise specified. Clinicopathological data were prospectively entered and clinical outcomes were regularly updated as part of database management. All patients underwent pancreatectomy for PDAC. Adjuvant therapy was offered to all patients as standard practice and the regimen was at the discretion of the treating oncologist based on the best available evidence at that time, provided patients recovered sufficiently after surgery and consented to receiving adjuvant therapy (*Table S1*). Completion of at least three cycles of adjuvant therapy was selected as a predefined threshold to indicate meaningful systemic exposure, reflecting real-world treatment patterns across the recruitment interval. For selected patients, neoadjuvant therapy (NAT) was used. The NAT regimens used were approved regimens at the discretion of the treating oncologist, except for a small cohort of patients who participated in the PRIMUS-002 trial (ISRCTN34129115, NCT04176952) as part of the Precision-Panc study. Ethical approval for the acquisition of data and biological material was obtained from the Human Research Ethics Committee at each participating institution (*Supplementary material*).

### Transcriptomic profiling

The molecular subtyping criteria were generated as part of the ICGC landmark study of PDAC^21^. RNA was extracted from bulk tumour and profiled using RNA sequencing and gene expression microarrays, as previously described^21^. Selecting patient samples to undergo sequencing was based on a number of factors, including cost, tissue quality, and tumour cellularity. All samples were fresh frozen upon collection. Tumours with cellularity <40% (249 patients) underwent gene expression microarray analysis, whereas RNA sequencing was performed for those tumour specimens with cellularity ≥40% (96 patients). Individual tumours were classified as belonging to either the squamous (basal-like) subtype or the classical pancreatic subtype, as previously described^21^. The classical pancreatic subtype encompassed the pancreatic progenitor, aberrantly differentiated endocrine exocrine (ADEX), and immunogenic subclasses described by Bailey *et al*.^21^. Differences in preselected squamous and classical genes, as well as gene programmes, were determined from RNA sequencing data. Differential gene expression was investigated for samples that underwent RNA sequencing using the ‘DESeq2’ package in R and plots were generated using the R package ‘ggplot2’.

### Statistical analysis

Statistical analysis was performed using SPSS^®^ (IBM, Armonk, NY, USA; version 29.0) and R 4.2.2 (The R Project for Statistical Computing, Vienna, Austria).

EOPC was defined as a diagnosis in patients aged <50 years, based on previous studies in PDAC and other cancers^10,11,14,16^. Categorical variables were compared using the chi-squared test. The Mann–Whitney *U* test was used to compare continuous variables. The principal outcomes measured were the duration of disease-specific survival (DSS) and disease-free survival (DFS), measured from the time of initial surgery or commencement of NAT. Differences in expression of individual genes or gene programmes between patient groups was assessed using the Wilcoxon rank test.

Patients alive at the time of follow-up, or who were lost to follow-up, were censored at the last clinical interaction. The last follow-up for patients still alive was in July 2024. Kaplan–Meier analysis was used to compare survival outcomes. To compare the length of survival between curves, a log rank test was performed. A Cox proportional hazards model was used for univariate analysis, to adjust for competing risk factors, and HRs with 95% confidence intervals are reported as estimates of the risk of DSS. Variables found to be significant in univariate analysis at *P* < 0.100 were included in multivariate analysis in a backwards stepwise fashion. The cumulative risk of recurrence was calculated using the Fine–Gray method of competing risk analysis using the ‘cmprsk’ and ‘bshazard’ packages in R. Patients with missing values for variables included in multivariable models were excluded from those specific analyses. The proportion of missing data for individual variables is reported in the appropriate table.

## Results

In total, 851 patients were identified who underwent pancreatic resection for PDAC. Of these, 68 (8.0%) had EOPC. The cohorts demonstrated clinical and pathological features that are typical for PDAC accrued over this interval (*Table 1*). Patients’ treatment and management was decided upon by a dedicated pancreatic multidisciplinary team. Adjuvant therapy and NAT were assigned as per local guidelines and the evidence base at the time (*Table S1*). Of the total patient cohort, 274 underwent transcriptomic analysis. Whilst 375 underwent germline mutation analysis, of whom 242 underwent transcriptomic analysis.

**Table 1:** Clinicopathological features of EOPC patients and late age-onset pancreatic cancer patients

Variables	All patients, *n* = 851	EOPC patients (aged <50 years), *n* = 68	Late age-onset pancreatic cancer patients (aged ≥50 years), *n* = 783	*P* (chi-squared)
**Sex**				
Male	451 (53.0)	34 (50.0)	417 (53.3)	
Female	400 (47.0)	34 (50.0)	366 (46.7	0.606
**Pathological T stage (AJCC 8th Edition)**				
T1	154 (18.1)	15 (22.1)	139 (17.8)	
T2	505 (59.3)	36 (52.9)	469 (59.9)	
T3	168 (19.7)	16 (23.5)	152 (19.4)	
Unknown	24 (2.8)	1 (1.5)	23 (2.9)	0.437
**Pathological N stage (AJCC 8th Edition)**				
N0	226 (26.6)	19 (27.9)	207 (26.4)	
N1	338 (39.7)	23 (33.8)	315 (40.2)	
N2	275 (32.3)	25 (36.8)	250 (31.9)	
Unknown	12 (1.4)	1 (1.5)	11 (1.4)	0.562
**Grade/tumour differentiation**				
Low	511 (60.0)	36 (52.9)	475 (60.7)	
High	279 (32.8)	28 (41.2)	251 (32.1)	
Unknown	61 (7.2)	4 (5.9)	57 (7.3)	0.092
**Margins (R1)**				
Clear	455 (53.5)	32 (47.1)	423 (54.0)	
Involved	375 (45.2)	35 (51.4)	350 (44.7)	
Not available	11 (1.3)	1 (1.5)	10 (1.3)	0.543
**Perineural invasion**				
Negative	126 (14.8)	12 (17.6)	114 (14.6)	
Positive	691 (81.2)	54 (79.4)	637 (81.4)	
Unknown	34 (4.0)	2 (2.9)	32 (4.1)	0.517
**Lymphovascular invasion**				
Negative	339 (39.8)	25 (36.8)	314 (40.1)	
Positive	430 (50.5)	35 (51.5)	395 (50.4)	
Unknown	82 (9.6)	8 (11.8)	74 (9.5)	0.695
**Adjuvant chemotherapy**				
No	264 (31.0)	15 (22.1)	249 (31.8)	
Yes	433 (50.9)	43 (63.2)	390 (49.8)	
Unknown	154 (18.1)	10 (14.7)	144 (18.4)	0.032
**NAT**				
No	719 (84.5)	59 (86.8)	660 (84.3)	
Yes	132 (15.5)	9 (13.2)	123 (15.7)	
Unknown	0 (0.0)	0 (0.0)	0 (0.0)	0.369
**Tumour location**				
Head	718 (84.4)	62 (91.2)	656 (83.8)	
Body/tail	123 (14.5)	6 (8.8)	117 (14.9)	
Unknown	10 (1.2)	0 (0.0)	10 (1.3)	0.104

Values are *n* (%) unless otherwise indicated. R1, microscopic resection margin involvement (<1 mm); NAT, neoadjuvant therapy.

### EOPC is associated with earlier recurrence after pancreatectomy

**Fig. 1:**
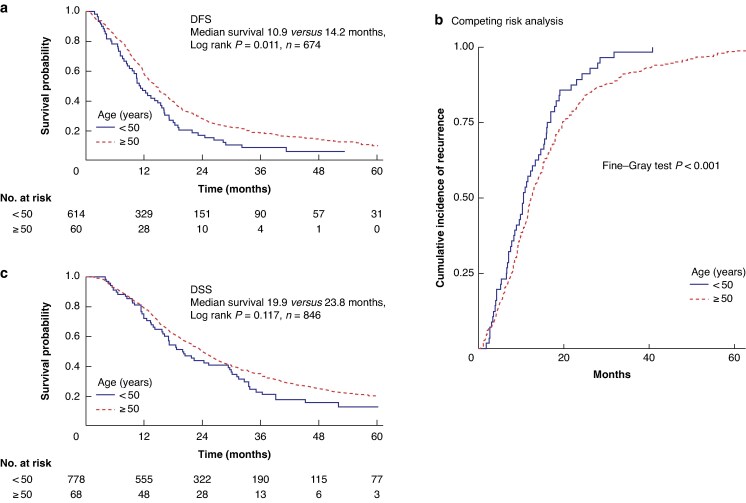
Overall survival and recurrence following pancreatectomy in EOPC **a** Kaplan–Meier curves demonstrating a difference in DFS between early- and late age-onset PDAC. **b** Fine–Gray competing risk analysis, demonstrating higher risk of early recurrence in EOPC. **c** Kaplan–Meier survival curves demonstrating similar DSS for early- and late age-onset PDAC. DFS, disease-free survival; DSS, disease-specific survival; PDAC, pancreatic ductal adenocarcinoma; EOPC, early-onset pancreatic cancer.

**Fig. 2:**
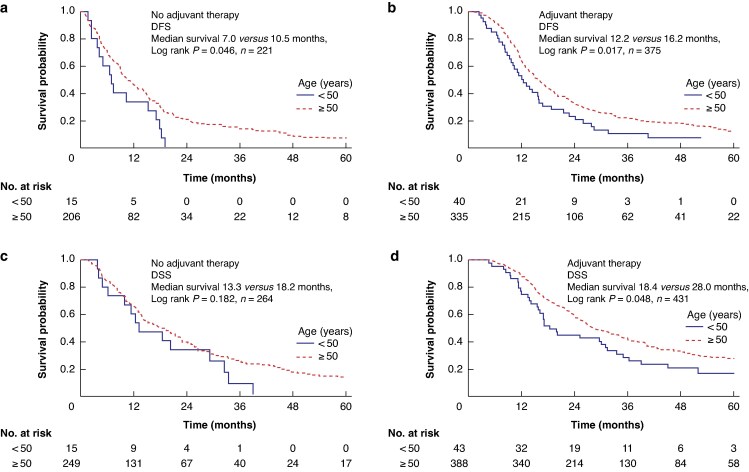
Differences in survival in EOPC stratified by adjuvant chemotherapy use **a** Kaplan–Meier survival curves demonstrating a difference in DFS between early- and late age-onset PDAC for patients who did not receive adjuvant chemotherapy. **b** Kaplan–Meier survival curves demonstrating a difference in DFS between early- and late age-onset PDAC for patients who did receive adjuvant chemotherapy. **c** Kaplan–Meier survival curves demonstrating a difference in DSS between early- and late age-onset PDAC for patients who did not receive adjuvant chemotherapy. **d** Kaplan–Meier survival curves demonstrating a difference in DSS between early- and late age-onset PDAC for patients who did receive adjuvant chemotherapy. DFS, disease-free survival; DSS, disease-specific survival; PDAC, pancreatic ductal adenocarcinoma.

In the identified cohort, EOPC was associated with earlier recurrence after pancreatectomy, with a median DFS of 10.9 *versus* 14.2 months (*P* = 0.011) (*Fig. 1a*). Considering death from other causes, a competing risk model using the Fine–Gray method demonstrated an increased risk of recurrence in the EOPC group compared with the late age-onset group (*P* < 0.001) (*Fig. 1b*). This was associated with a non-significant trend of worse DSS (median 19.9 *versus* 23.8 months; *P* = 0.117) (*Fig. 1c*). For those with accurate chemotherapy data, EOPC patients were significantly more likely to start adjuvant chemotherapy (74% *versus* 61%; *P* = 0.049) and more likely to complete at least three cycles of adjuvant chemotherapy (67% *versus* 51%; *P* = 0.029). When comparing the outcomes of those who received adjuvant chemotherapy, the EOPC group was associated with worse DFS (12.2 *versus* 16.2 months; *P* = 0.017) (*Fig. 2a,b*) and DSS (18.4 *versus* 28.0 months; *P* = 0.048) (*Fig. 2c,d*) when completing adjuvant therapy. To address the influence of adjuvant therapy on long-term DSS in the EOPC group, multivariate survival analysis, including all validated prognostic variables, was performed (*Table 2*). This demonstrated that EOPC was an independent predictor of worse survival (HR 1.52 (95% c.i. 1.09 to 2.10); *P* = 0.012). Pathological N stage (HR 1.98 (95% c.i. 1.47 to 2.69); *P* < 0.001), pathological T stage (HR 1.84 (95% c.i. 1.31 to 2.57); *P* < 0.001), lymphovascular invasion (HR 1.49 (95% c.i. 1.20 to 1.84); *P* < 0.001), high tumour grade (HR 1.52 (95% c.i. 1.24 to 1.87); *P* < 0.001), positive resection margin (HR 1.30 (95% c.i. 1.06 to 1.60); *P* < 0.001), and adjuvant therapy (HR 0.53 (95% c.i. 0.43 to 0.65); *P* < 0.001) were all independent predictors within the multivariate model, as expected from previous studies (*Table 2*). Separate multivariate models for EOPC and late age-onset PDAC demonstrated that DSS for EOPC patients was independently dependent on pathological N stage (*P* = 0.023), lymphovascular invasion (*P* = 0.012), and adjuvant chemotherapy (*P* < 0.001), but, in contrast to late age-onset PDAC, DSS was not dependent on other factors such as margin status, pathological T stage, and tumour grade (*Tables S2*, *S3*). In addition, EOPC was not associated with any clinicopathological features that would explain worse DFS (*Table 1*), including pathological N stage (*P* = 0.562). These findings suggest that EOPC is associated with worse outcomes after pancreatic resection, independent of validated prognostic clinicopathological variables.

**Table 2:** Multivariate analysis for DSS

	HR (95% c.i.)	*P*
**Pathological N stage**		
N0	Reference category	
N1	1.60 (1.20,2.12)	0.001
N2	1.98 (1.47,2.69)	<0.001
**Pathological T stage**		
T1	Reference category	
T2	1.26 (0.94,1.68)	0.123
T3	1.84 (1.31,2.57)	<0.001
Lymphovascular invasion	1.49 (1.20,1.84)	<0.001
Perineural invasion	1.28 (0.94,1.74)	0.121
Grade (high)	1.52 (1.24,1.87)	<0.001
Margin (R1 < 1 mm)	1.30 (1.06,1.60)	<0.001
Adjuvant therapy	0.53 (0.43,0.65)	<0.001
NAT	0.90 (0.65,1.23)	0.500
Tumour location (body/tail)	1.06 (0.78,1.1.44)	0.733
EOPC	1.52 (1.09,2.10)	0.012

All patients (final model, *n* = 851). DSS, disease-specific survival; NAT, neoadjuvant therapy; EOPC, early-onset pancreatic cancer.

### EOPC is associated with the squamous subtype and an adverse transcription profile

**Fig. 3:**
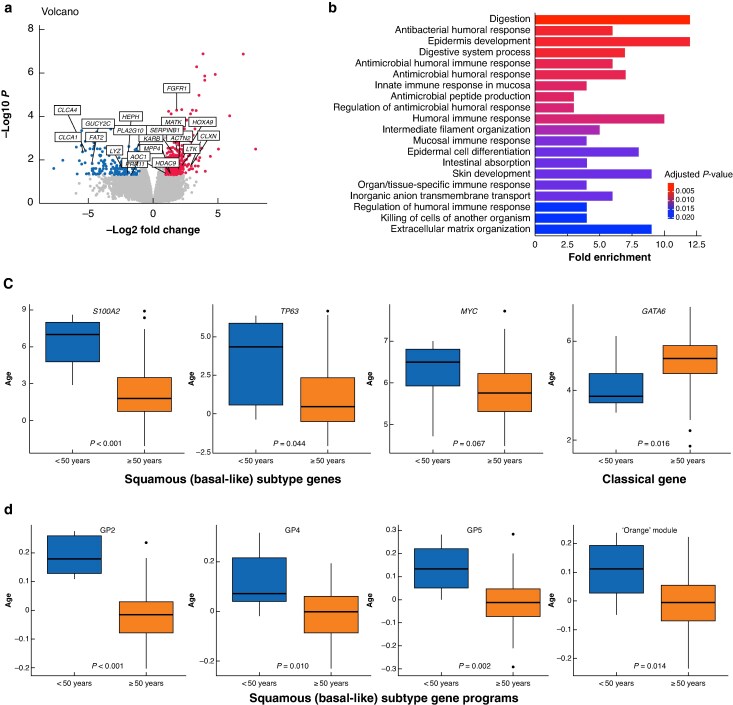
Molecular differences between EOPC and late age-onset PC **a** Volcano plot of differentially expressed genes for early- and late age-onset PDAC. The top 20 differentially expressed genes are highlighted. **b** Gene ontology terms for significantly down-regulated expressed genes in EOPC. **c** Normalized expression of squamous and classical subtype genes in early- *versus* late age-onset PDAC. **d** Differential expression of gene programmes that define the squamous subtype in EOPC. GP, gene programme; PDAC, pancreatic ductal adenocarcinoma; EOPC, early-onset pancreatic cancer.

Of the patients who underwent germline mutation analysis, 23 (6.1%) had EOPC. Of these patients, only three (13%) had a germline mutation, whereas germline aberrations were present in 17% of patients with late age-onset PDAC (*Table S5*). To investigate any molecular factors underlying the clinical findings described above, the APGI transcriptomic cohort was interrogated. There were significant differences in gene expression (393 genes) between the two groups (*Fig. 3a*). To further identify potential biological differences between groups, the differentially expressed genes were converted into gene ontology terms (*Fig. 3b*). This demonstrated down-regulation in the EOPC group of gene programmes associated with innate and adaptive immune responses, as well as extracellular matrix organization (*Fig. 3b*).

Genes associated with the squamous (basal-like) subtype of PDAC were associated with EOPC in the RNA sequencing cohort (*Fig. 3c*). Expression of *MYC* (*P* = 0.067), *S100A2* (*P* < 0.001), and *TP63* (*P* = 0.044) was enriched in the EOPC group; these genes are highly expressed in the squamous subtype of PDAC and are associated with poor prognoses (*Fig. 3c*). In contrast, *GATA6*, a key gene associated with the classical pancreatic subtype, was down-regulated in the EOPC group (*P* = 0.016). The squamous subtype was enriched in the EOPC group (42% *versus* 28%; *P* = 0.145), but this was not statistically significant (*Table S4*). When comparing gene programmes, described by Bailey *et al*.^21^ that are associated with PDAC molecular subtypes, there was significant enrichment in the EOPC group of gene programmes that are associated with the squamous subtype (*Fig. 3d* and *Fig. S1*): gene programme 2 (squamous differentiation, inflammation, and *TP63* signalling), gene programme 4 (cell proliferation), and gene programme 5 (*MYC* activation). In contrast, the late age-onset group was associated with gene programmes that are associated with the classical pancreatic subtype (*Fig. S1*).

To determine whether these transcriptomic differences translated into changes in the tumour microenvironment, previously generated immunohistochemistry and immune deconvolution data were interrogated. The EOPC group was enriched with regard to CD68^+^ macrophages, but this was not statistically significant (*P* = 0.088) (Fig. *S2a*). There were no differences in overall expression of CD8^+^ T cells (*P* = 0.162), CD3^+^ T cells (*P* = 0.946), or CD163^+^ macrophages (*P* = 0.551) (Fig. *S2*a). Using the CIBERSORT deconvolution package, there was a statistically significant increase in expression of naïve B cells (*P* = 0.047) and follicular helper T cells (*P* = 0.038) in the late age-onset group; however, these are both expressed at such low levels in the cohort, that the significance is difficult to interpret (Fig. *S2*b). There appeared to be significant enrichment of M2 macrophages in the EOPC group; however, this again failed to reach statistical significance (*P* = 0.078). Overall, these findings support the notion that molecular differences appear to exist in EOPC *versus* late age-onset disease, with an association of molecular factors that underlie the squamous subtype. This does not translate into significant differences in the quantity of immune cells present within the tumour microenvironment, but functional interactions and complex microenvironment analyses were not performed.

## Discussion

There is growing concern regarding the increase in early-onset gastrointestinal cancers, with pancreatic cancer being no exception. In the present study, EOPC is demonstrated to be associated with earlier disease recurrence after pancreatectomy, despite increased administration of multimodality treatment in the form of adjuvant chemotherapy. There was no association with standard clinical and pathological features to explain these differences. To understand this phenomenon, transcriptomic and tumour microenvironment features that may be responsible for biological differences between EOPC and late age-onset disease were investigated. The results suggest that EOPC is associated with molecular features that are associated with the squamous (basal-like) molecular subtype of PDAC.

Previous studies have investigated the clinical outcomes of EOPC compared with those of late age-onset disease^15,16^. In a large cohort of 136 young patients with PDAC, good long-term outcomes of resected patients with localized PDAC were reported; however, these patients represented only a minority of the overall cohort^22^. Similarly, a study of consecutive patients presenting with PDAC of all stages found that EOPC patients had similar overall survival compared with late age-onset PDAC patients^15^. A very recent study of >1600 patients with PDAC, of whom 112 had EOPC, demonstrated improved outcomes for EOPC^16^. This finding was conserved across resected, locally advanced, and metastatic patients^16^. However, a notable finding of the study by Mendis *et al*.^16^ was that the late age-onset group was significantly more frail than the EOPC group and this may have influenced patients’ fitness to receive any treatment. Poor performance status, higher Charlson co-morbidity index, and weight loss were all features of the late age-onset cohort (aged >50 years)^16^. These are all validated variables that determine survival and initiation of treatment, and may explain the better outcomes seen in the EOPC group. Unfortunately, these variables were not recorded in the data set for the present study, so a direct comparison was not possible. However, a significantly increased utilization of adjuvant chemotherapy was observed in the EOPC group, which is likely to reflect the increased proportion of functional recovery and better performance status after surgery for younger patients. Despite this, there was no improved survival in the EOPC group, with earlier recurrence after surgery. When accounting for other prognostic variables in the multivariate model, early age-onset of disease was an independent predictor of worse cancer-specific survival in the cohort of the present study. Furthermore, for patients who received multimodal therapy, which included surgery and systemic chemotherapy, both DSS and DFS were worse in the EOPC group. Thus, it can be concluded that, in a cohort of patients for whom co-morbidities and frailty are balanced out, so that all undergo surgery and systemic therapy for PDAC, early age of onset is a poor prognostic factor for survival after pancreatectomy. Clinical and pathological features such as pathological N stage, pathological T stage, margin status, tumour location, tumour grade, and other histological features did not explain these differences.

Several groups have defined reproducible molecular subtypes of PDAC that have since been demonstrated to be clinically relevant^18,21,23–25^. Despite not being statistically significant, the EOPC cohort that underwent transcriptomic profiling in the present study was heavily enriched with regard to the squamous subtype. Thus, the individual genes and gene programmes that are associated with the squamous subtype were investigated and the EOPC group was found to have significantly higher expression of these. Gene programmes involved in proliferation, squamous differentiation, inflammation, and metabolic reprogramming were overexpressed in the EOPC group. When performing differential gene expression and subsequent gene ontology analysis, EOPC had relative down-regulation of gene programmes associated with innate and adaptive immune responses. These findings suggest that, for the EOPC cohort in the present study, molecular features of more aggressive disease were present and these possibly explain the worse clinical outcomes that were observed. This did not translate into any significant differences in the composition of the tumour microenvironment in the EOPC group compared with the late age-onset group. Yet, there was increased, but statistically non-significant, gene and protein expression associated with macrophages in the EOPC group. Similar findings were seen in a previous study where EOPC was enriched with regard to M2 macrophages after deconvolution of gene expression data^14^. The statistically significant differences seen in the study by Ogobuiro *et al.*^14^, compared with statistically non-significant differences seen in the present study, may reflect the increased number of patients included (284 EOPC patients). The immune landscape and tumour microenvironment in PDAC is extremely complex and cumulative infiltration of immune cells does not accurately portray the underlying immune biology. Thus, more complex spatial interaction analyses are required to determine whether any differences in immune function exist in the EOPC patients.

Few studies have been able to discern the molecular heterogeneity that exists between EOPC and late age-onset disease^14,22,26,27^. Ogobuiro *et al.*^14^ and Tsang *et al*.^27^ found enrichment of genes involved in the extracellular matrix and inflammatory response in EOPC. A different study, by Ben-Aharon *et al.*^26^, found that EOPC was associated with transforming growth factor-β signalling and, similar to the study by Ogobuiro *et al.*^14^ , found a higher proportion of *KRAS* wild type PDAC in EOPC.

The present study has several limitations. An age cut-off of <50 years for EOPC was used, which is informed by previous studies^14,15,28–30^, but may not distinguish subtle molecular differences in very young patients (those aged <40 years). Second, the study only included surgically resected patients, thus representing only a minority of patients with PDAC. Thus, molecular differences may be under-represented and need to be explored in contemporary metastatic and locally advanced cohorts of early- and late age-onset PDAC. In addition, germline mutational analysis was not performed for all patients. Thereby, potential clinical and molecular differences in the groups could be secondary to inherited mutations. However, in the cohort that did undergo germline analysis, there was no association of EOPC with inherited mutations. In addition, the vast majority of the molecular cohort did have germline analysis (88%), suggesting that the low frequency of inherited mutations in EOPC in this cohort is unlikely to have influenced the findings.

The incidence of EOPC is on the rise, alongside that of other gastrointestinal cancers, and thus these data further add to the limited literature on the molecular taxonomy of what remains a rare cancer in younger patients^14,22,26,27^. The findings of this study have important clinical implications. Younger patients with pancreatic cancer are often presumed to have more favourable tumour biology and superior treatment tolerance. However, the present study demonstrates that early age-onset disease is associated with earlier recurrence and enrichment of aggressive squamous-associated transcriptional programmes, independent of conventional clinicopathological factors. These results suggest that age at diagnosis may represent a clinically relevant biological stratifier rather than merely a demographic variable. EOPC should not be assumed to confer a prognostic advantage after resection and may warrant closer postoperative surveillance, early optimization of systemic therapy, and prioritization for clinical trial enrolment. Future studies should explore whether intensified or biologically guided treatment strategies may improve outcomes in this subgroup. This will require multinational collaboratives set up to interrogate these using the genome, transcriptome, immune, and exposome to make clinically relevant discoveries, with the aim to start improving outcomes for this deadly cancer.

## Supplementary Material

znag032_Supplementary_Data

## Data Availability

Patient data are not available for sharing due to confidentiality. All sequencing data are available from the International Cancer Genome Consortium (ICGC) portal (https://docs.icgc-argo.org/docs/data-access/icgc-25k-data).
