# Supplementary material for: Clinical and molecular landscape of surgically resected early onset pancreatic cancer
Source: Br J Surg. 2026 May 7;113(5):znag032. doi: 10.1093/bjs/znag032 (PMC13151028; doi:10.1093/bjs/znag032)
Supplement: znag032_Supplementary_Data [file znag032_supplementary_data.docx]

**Supplementary data**

**The clinical and molecular landscape of surgically resected early age-onset pancreatic cancer**

Stephan B Dreyer^1,2,3 MD, PhD^, Adam Bryce^1^, Fieke Froeling^1,4^, Shannon Jackson^2^, Australian Pancreatic Cancer Genome Initiative^5^, Leonor Santana^1^, Euan J. Dickson^2 MD^, Maria Coats^2^, Colin McKay^2^, David Holroyd^2^, Andrew V. Biankin^1,2 MD, PhD^, Nigel B Jamieson^1,2 MD, PhD^, David K. Chang^1,2 MD, PhD^

^1^Wolfson Wohl Cancer Research Centre, School of Cancer Sciences, University of Glasgow, Estate, Switchback Road, Bearsden, Glasgow, United Kingdom ; ^2^West of Scotland Pancreatic Unit, Glasgow Royal Infirmary, Glasgow, United Kingdom; ^3^Department of HPB/Transplant Surgery, Royal Infirmary of Edinburgh, Edinburgh, United Kingdom; ^4^Department of Oncology, Beatson West of Scotland Cancer Centre, Glasgow, United Kingdom; ^5^The Kinghorn Cancer Centre, Darlinghurst and a Institute of Medical Research, Sydney, AUSTRALIA.

**Corresponding authors:**

**Stephan B Dreyer**

Wolfson Wohl Cancer Research Centre,

School of Cancer Sciences, University of Glasgow,

Garscube Estate, Switchback Road,

Bearsden, Glasgow Scotland G61 1BD

Email: [stephan.dreyer@glasgow.ac.uk](mailto:stephan.dreyer@glasgow.ac.uk)

X (Twitter ID) : stephan_dreyer

ORCID ID : 0000-0001-6134-2096

**David K. Chang**

Wolfson Wohl Cancer Research Centre,

Shool of Cancer Sciences, University of Glasgow,

Garscube Estate, Switchback Road,

Bearsden, Glasgow Scotland G61 1BD

Tel: +44 141 330 7589 Fax: +44 141 330 5834

Email: [david.chang@glasgow.ac.uk](mailto:david.chang@glasgow.ac.uk)

# Australian Pancreatic Cancer Genome Initiative (APGI)

# Contact APGI: [research@pancreaticcancer.net.au](mailto:research@pancreaticcancer.net.au)

# List of APGI researchers

**Garvan Institute of Medical Research** Amber L. Johns^1^, Anthony J Gill^1,5^, Lorraine A. Chantrill^1,22^, Paul Timpson^1,^ Angela Chou^1,5^, Marina Pajic^1^, Tanya Dwarte^1^, David Herrmann^1^, Claire Vennin^1^, Thomas R Cox^1^, Brooke Pereira^1^, Shona Ritchie^1^, Daniel A Reed^1^, Cecilia R Chambers^1^, Max Nobis^1^, Gloria Jeong^1^, Ruth J. Lyons^1^, Nicola Blackburn^1^, Adnan Nagrial^1^, Sean Porazinski^1^, Diego Chacon Fajardo^1^, Alice Russo^1^. **QIMR Berghofer Medical Research Institute** Nicola Waddell^2^, John V. Pearson^2^, Katia Nones^2^, Felicity Newell^2^, Venkateswar Addala^2^, Oliver Holmes^2^, Conrad Leonard^2^, Scott Wood^2^. **University of Melbourne, Centre for Cancer Research** Sean M. Grimmond^3^, Oliver Hofmann^3^. **Royal North Shore Hospital** Jaswinder S. Samra^5^, Nick Pavlakis^5,^ Jennifer Arena^5^, Hilda A. High^5^, Anubhav Mittal^5^. **Bankstown Hospital** Ray Asghari^6^, Neil D. Merrett^6^, Amitabha Das^6^. **Liverpool Hospital** Peter H. Cosman^7^, Kasim Ismail^7^. **St Vincent’s Hospital** Alina Stoita^8^, David Williams^8^, Allan Spigellman^8^**. Westmead Hospital** Duncan McLeod^9^, Judy Kirk^9^. **Royal Prince Alfred Hospital, Chris O’Brien Lifehouse** James G. Kench^10^, Peter Grimison^10^, Charbel Sandroussi^10^, Annabel Goodwin^7,10^. **Prince of Wales Hospital** R. Scott Mead^1,11^, Katherine Tucker^11^, Lesley Andrews^11^. **Fiona Stanley Hospital** Michael Texler^12^, Cindy Forrest^12^, Mo Ballal^12,13^, David Fletcher^12^**. St John of God Healthcare** Maria Beilin^13^, Kynan Feeney^13^ Krishna Epari^13^ Sanjay Mukhedkar^13^. **Epworth HealthCare** Nikolajs Zeps^23^. **Royal Adelaide Hospital** Nan Q Nguyen^14^, Andrew R. Ruszkiewicz^14^, Chris Worthley^14^. **Flinders Medical Centre** John Chen^15^, Mark E. Brooke-Smith^15^, Virginia Papangelis^15^. **Envoi Pathology** Andrew D. Clouston^16^. **Princess Alexandra Hospital** Andrew P. Barbour^17^, Thomas J. O’Rourke^17^, Jonathan W. Fawcett^17^, Kellee Slater^17^, Michael Hatzifotis^17^, Peter Hodgkinson^17^. **Austin Hospital** Mehrdad Nikfarjam^18^. **Johns Hopkins Medical Institutes** James R. Eshleman^19^, Ralph H. Hruban^19^, Christopher L. Wolfgang^19^. **ARC-Net Centre for Applied Research on Cancer** Aldo Scarpa^20^, Rita T. Lawlor^20^, Vincenzo Corbo^20^, Claudio Bassi^20^. **University of Glasgow** Andrew V Biankin ^21^, Nigel B. Jamieson^21^ David K. Chang^1, 21,^ Stephan B. Dreyer^21^.

^1^The Kinghorn Cancer Centre, Garvan Institute of Medical Research, 370 Victoria Street, Darlinghurst, Sydney, New South Wales 2010, Australia.

^2^QIMR Berghofer Medical Research Institute, 300 Herston Rd, Herston, Queensland 4006, Australia.

^3^University of Melbourne, Centre for Cancer Research, Victorian Comprehensive Cancer Centre, 305 Grattan Street, Melbourne, Victoria 3000, Australia.

^4^ Institute for Molecular Bioscience, University of QLD, St Lucia, Queensland 4072, Australia.

^5^Royal North Shore Hospital, Westbourne Street, St Leonards, New South Wales 2065, Australia.

^6^Bankstown Hospital, Eldridge Road, Bankstown, New South Wales 2200, Australia.

^7^Liverpool Hospital, Elizabeth Street, Liverpool, New South Wales 2170, Australia.

^8^ St Vincent’s Hospital, 390 Victoria Street, Darlinghurst, New South Wales, 2010 Australia.

^9^Westmead Hospital, Hawkesbury and Darcy Roads, Westmead, New South Wales 2145, Australia.

^10^Royal Prince Alfred Hospital, Missenden Road, Camperdown, New South Wales 2050, Australia.

^11^Prince of Wales Hospital, Barker Street, Randwick, New South Wales 2031, Australia.

^12^Fiona Stanley Hospital, 11 Robin Warren Dr, Murdoch WA 6150

^13^ St John of God Healthcare, 12 Salvado Road, Subiaco, Western Australia 6008, Australia

^14^ Royal Adelaide Hospital, North Terrace, Adelaide, South Australia 5000, Australia.

^15^ Flinders Medical Centre, Flinders Drive, Bedford Park, South Australia 5042, Australia.

^16^ Envoi Pathology, 1/49 Butterfield Street, Herston, Queensland 4006, Australia.

^17^ Princess Alexandra Hospital, 199 Ipswich Rd, Woolloongabba QLD 4102

^18^ Austin Hospital, 145 Studley Road, Heidelberg, Victoria 3084, Australia.

^19^ Johns Hopkins Medical Institute, 600 North Wolfe Street, Baltimore, Maryland 21287, USA.

^20^ ARC-NET Center for Applied Research on Cancer, University of Verona, Via dell’Artigliere, 19 37129 Verona, Province of Verona, Italy.

^21^ Wolfson Wohl Cancer Research Centre, Institute of Cancer Sciences, University of Glasgow, Garscube Estate, Switchback Road, Bearsden, Glasgow, Scotland G61 1BD, United Kingdom.

^22^ Wollongong Hospital, Illawarra and Shoalhaven Local Health District, Loftus Street, Wollongong NSW 2500.

^23^ Epworth HealthCare, 89 Bridge Rd, Richmond VIC 3121, Australia

**Ethics approval numbers**

- Sydney Local Health District (RPA Zone) : Approval number X16-0293
- University of Melbourne Health Sciences Human Ethics Subcommittee: Approval Number 1748955.
- North Shore Private Hospital Ethics Committee: Approval number NSPHEC 2016-016.
- Garvan Institute of Medical Research Approval number 1627.
- West of Scotland Research Ethics Service (WoSRES) committee, NHS Greater Glasgow and Clyde-Molecular profiling of pancreatic cancer for improved prediction of Survival. Research Ethics Committee reference number: 07/S0704/26
- Ethics approval for Royal North Shore Hospital cohort was obtained from Northern Sydney Local Health District Human Research and Ethics Committee (NSLHD HREC HREC/16/HAWKE/105).

**Supplementary Tables**

| **Supplementary table 1: Adjuvant chemotherapy regimens**  **(n = 432)** | |
| --- | --- |
| Regimen | No (%) |
| **Gemcitabine-based therapy**    Gemcitabine monotherapy    Gemcitabine + Capecitabine    Gemcitabine + Erlotinib    Gemcitabine + Nab-paclitaxel    Carboplatin + Gemcitabine  **Subtotal, gemcitabine-based** | 288 (66.7)  41 (9.5)  2 (0.5)  4 (0.9)  1 (0.2)  **336 (77.7)** |
| **FOLFIRINOX-based therapy**    FOLFIRINOX (standard)    Modified FOLFIRINOX  **Subtotal, FOLFIRINOX-based** | 16 (3.7)  7 (1.6)  **23 (5.3)** |
| **Fluoropyrimidine-based therapy**    5-Fluorouracil (5-FU) monotherapy    5-FU + Folinic acid    FOLFOX  **Subtotal, fluoropyrimidine-based** | 21 (4.9)  6 (1.4)  1 (0.2)  **28 (6.5)** |
| Capecitabine monotherapy  Cisplatin + Etoposide | 2 (0.5)  1 (0.2) |
| **Unknown** | **42 (9.7)** |

| **Supplementary Table 2:**  multivariate analysis – Late onset  (final model, n = 784) | | |
| --- | --- | --- |
|  | **Multivariate Analysis** | |
|  | **HR (95% CI)** | ***P*** |
| **N-stage N0**  **N1**  **N2** | REF  1.50 (1.12 – 2.02)  1.96 (1.43 – 2.69) | 0.007  <0.001 |
| **T-stage T1**  **T2**  **T3** | REF  1.26 (0.93 – 1.72)  1.90 (1.33 – 2.73) | 0.136  <0.001 |
| **Lymphovascular Invasion** | 1.46 (1.17 – 1.84) | 0.001 |
| **Perineural invasion** | *1.12 (0.80 – 1.56)* | *0.507* |
| **Grade (High grade)** | 1.60 (1.29 – 1.99) | <0.001 |
| **Margin (R1 = 1mm)** | 1.35 (1.09 – 1.67) | 0.006 |
| **Adjuvant Therapy** | 0.55 (0.44 – 0.69) | <0.001 |
| **Neoadjuvant therapy** | *0.88 (0.64 – 1.22)* | *0.445* |
| **Tumour Location (body/tail)** | *1.11 (0.81 – 1.52)* | *0.526* |

**Abbreviations:** R1, microscopic resection margin involvement (< 1 mm); T, tumour stage; N, nodal stage;

| **Supplementary Table 3:**  multivariate analysis – EOPC  (final model, n = 68) | | |
| --- | --- | --- |
|  | **Multivariate Analysis** | |
|  | **HR (95% CI)** | ***P*** |
| **N-stage N0**  **N1**  **N2** | REF  2.14 (0.85 – 5.40)  2.95 (1.16 – 7.51) | 0.108  0.023 |
| **T-stage T1**  **T2**  **T3** | *REF*  *0.69 (0.27 – 1.79)*  *1.06 (0.37 – 3.04)* | *0.444*  *0.916* |
| **Lymphovascular Invasion** | 2.53 (1.23 - 5.18) | 0.012 |
| **Perineural invasion** | 2.15 (0.88 – 5.27) | 0.095 |
| **Grade (High grade)** | *0.89 (0.34 – 2.32)* | *0.810* |
| **Margin (R1 = 1mm)** | *0.60 (0.28 – 1.29)* | *0.193* |
| **Adjuvant Therapy** | 0.21 (0.08 – 0.53) | <0.001 |
| **Neoadjuvant therapy** | *0.66 (0.10 – 4.25)* | *0.662* |
| **Tumor Location (body/tail)** | *0.72 (0.17 – 3.08)* | *0.654* |

**Abbreviations:** EOPC, early age-onset pancreatic cancer, R1, microscopic resection margin involvement (< 1 mm); T, tumour stage; N, nodal stage;

| **Supplementary Table 4: The association of molecular subtype with EOPC** | | |
| --- | --- | --- |
| ***P = 0.145*** | **< 50** | **≥ 50** |
| **Classical** | 11 (58%) | 184 (72%) |
| **Squamous** | 8 (42%) | 71 (28%) |

| **Supplementary Table 5: Germline mutations in sequenced cohort (*n* = 375)** | | |
| --- | --- | --- |
| ***P = 0.575*** | **Gene Mutation** | **No of patients (%)** |
| **EOPC** | *FANCL*  *RAD51D*  *POLH* | 1 (4)  1 (4)  1 (4) |
| **LOPC** | *ATM*  *BLM*  *BRCA1*  *BRCA2*  *BLM*  *CTR9*  *BUB1B*  *CKDN2A*  *CHEK2*  *ERCC2*  *ERCC5*  *FANCA*  *FANCC*  *FANCE*  *FANCI*  *FANCL*  *FANCM*  *LIG4*  *MAP3K6*  *MUTYH*  *NBN*  *PALB2*  *PMS2*  *RET*  *RTEL1*  *SBDS*  *SEMA4A*  *STK11*  *TERT*  *VHL*  *XPC* | 4  1  3  9  3*  1*  1  2  1  2  1  2  1  1  1  2  1  1  2  3  7  3  1  1  1  5  1  1  4*  1  1 |

**Abbreviations:** EOPC, early age-onset pancreatic cancer, LOPC, late age-onset pancreatic cancer.

*** Mutation co-exist in presence of additional mutation**

**Supplementary Figures:**

**Supplementary Figure 1**

**
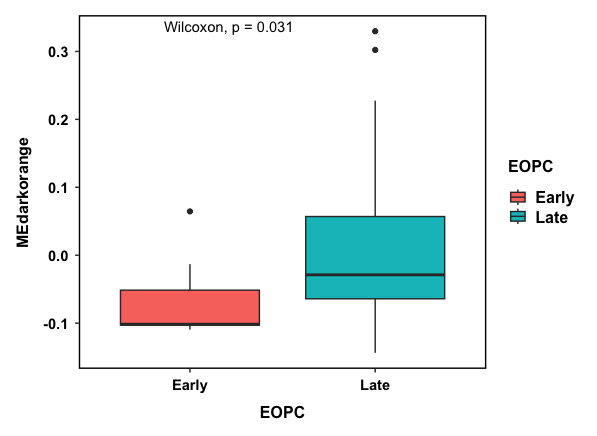

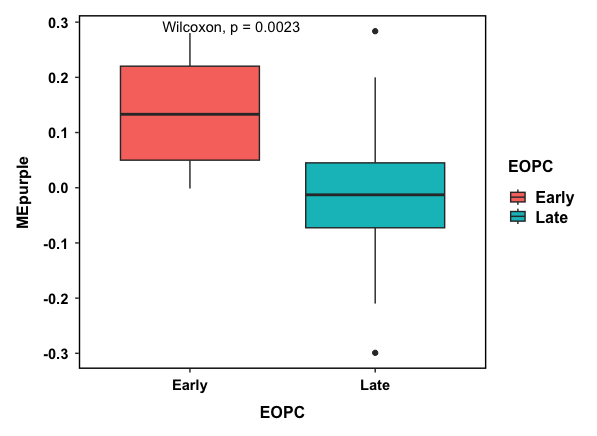

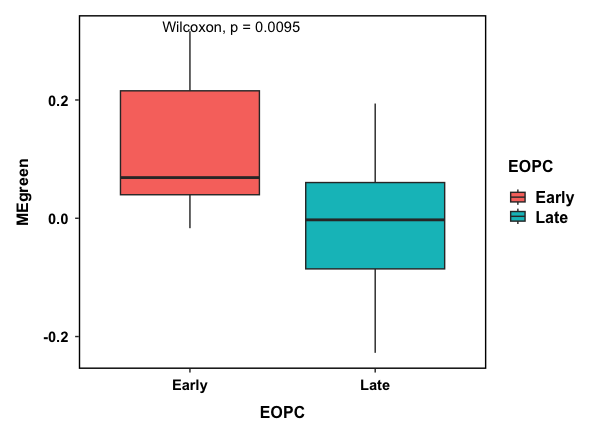

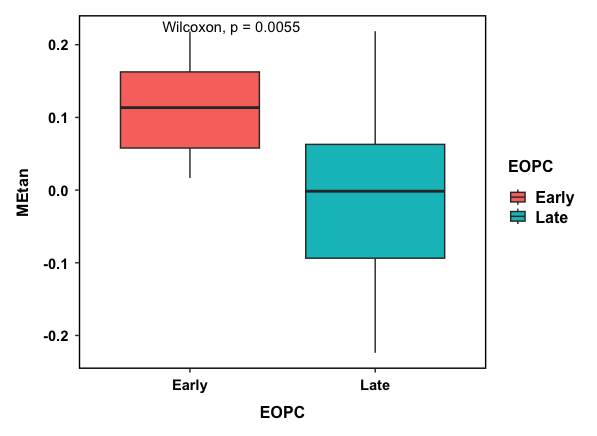

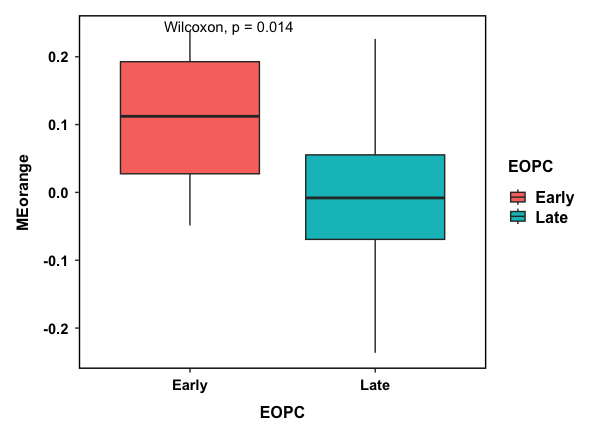

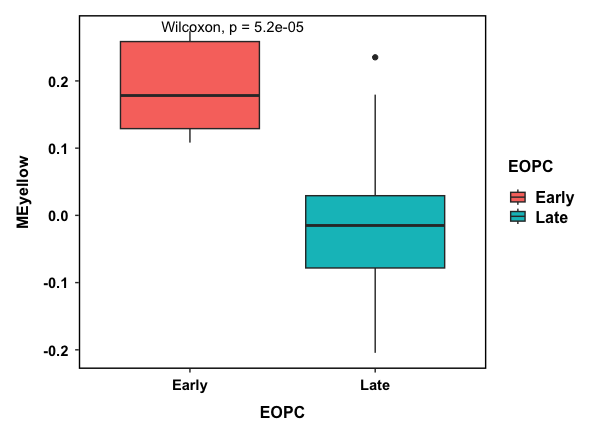

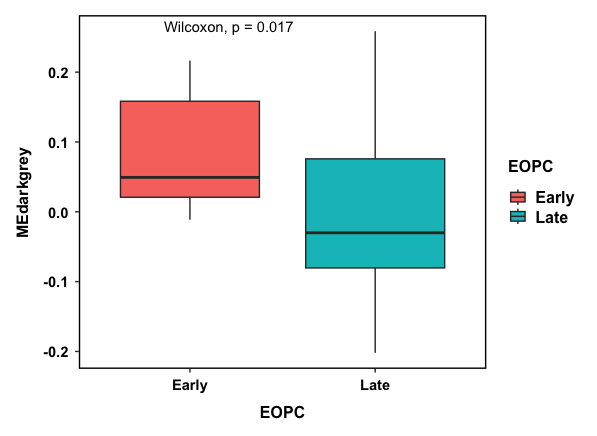

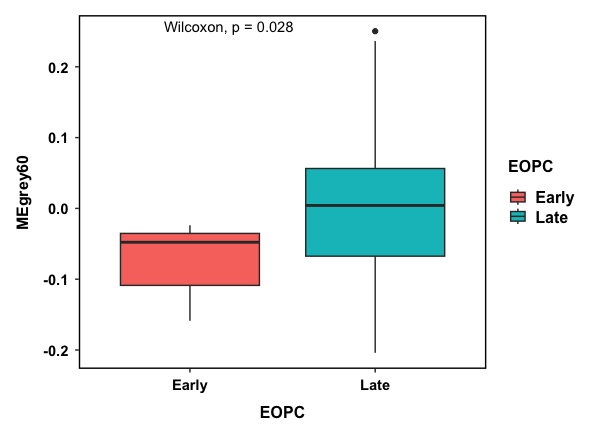

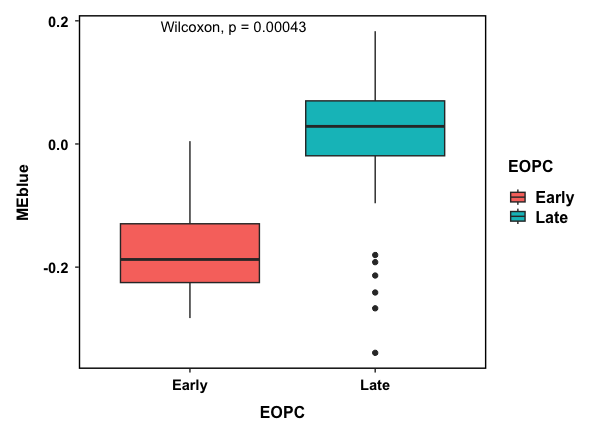
**

**Supplementary Figure 1:** The association of EOPC (< 50 yo) with gene programs as defined by Bailey *et al.*^1^*.*

**Supplementary Figure 2**

**
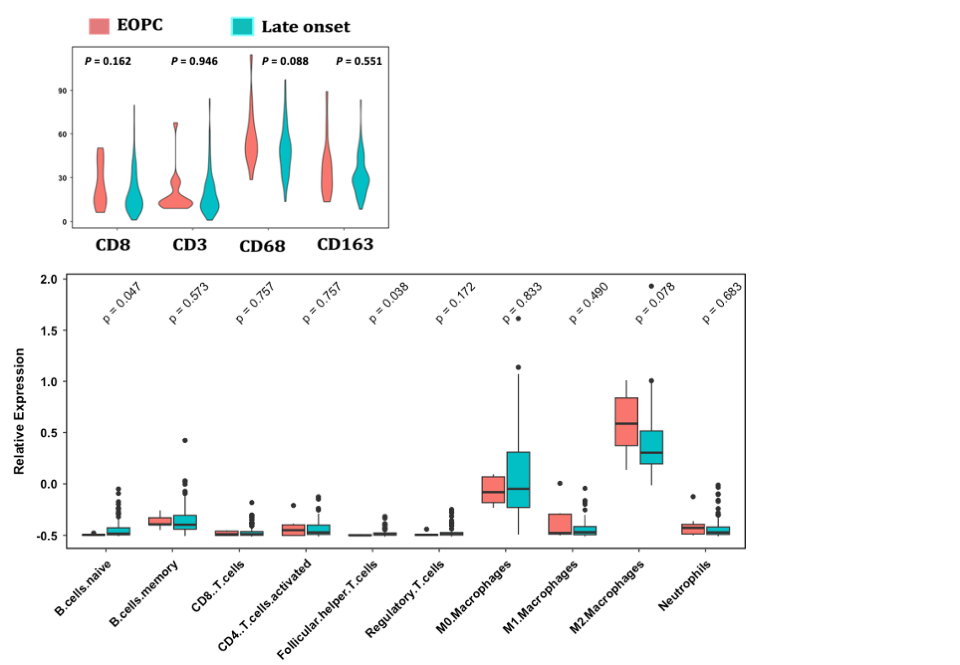
**

**B**

**A**

**Supplementary Figure 2: a)** Mean histoscores of Immunohistochemistry analysis of immune cells in early versus late onset PDAC. **B)** Immune deconvolution of RNA sequencing data comparing early versus late onset PDAC.

Reference:

1. Bailey, P. *et al.* Genomic analyses identify molecular subtypes of pancreatic cancer. *Nature* **531**, 47–52 (2016).
